# Glutathione S-transferase M1 and T1 polymorphisms and the risk of mild hepatotoxicity induced by carbamazepine in a tunisian population study

**DOI:** 10.1186/s12883-018-1013-8

**Published:** 2018-03-09

**Authors:** Chahra Chbili, Anis Hassine, Neila Fathallah, Manel Nouira, Salma Naija, Sofiene Ben Ammou, Saad Saguem

**Affiliations:** 1 Metabolic Biophysics, Professional Toxicology and Applied Environmental Laboratory, Department of Biophysics, Medicine Faculty of Sousse, Sousse University, Avenue Mohamed Karoui, 4002 Sousse, Tunisia; 2 Neurology Department of Central Hospital University (CHU), Sousse University, 4002 Sousse, Tunisia

**Keywords:** Glutathione-S-transferase M1, Glutathione-S-transferase T1, Epilepsy, Genetic polymorphisms, Carbamazepine

## Abstract

**Background:**

The aim of this study was to evaluate whether the glutathione S-transferase M1 (GSTM1) and T1 (GSTT1) null alleles may contribute to carbamazepine-induced hepatotoxicity.

**Methods:**

A cross-sectional prospective study was conducted to identify the frequency distribution of GSTM1 and GSTT1 alleles in 129 Tunisian epileptic patients treated with carbamazepine. Null alleles were determined using a Polymerase Chain Reaction. Serum alanine aminotransferase (ALT) and aspartate aminotransferase (AST) were measured by standard methods.

**Results:**

Our results showed that the frequencies of GSTM1 (−) null allele and GSTT1 null (−) allele were 74.4 and 17.8% respectively. The ALT and AST levels were elevated in 46 (35.7%) and 33 (25.6%) cases. The mean values of ALT and AST were approximately 1.32 and 3.61 times higher than the upper limit of normal levels, respectively. The values of ALT and AST were significantly higher in GSTM1 (−) allele than in GSTM1 (+) (*p* = 10^−3^.and 0.004, respectively).

The level of ALT was significantly higher in combination of GSTM1 (−)/T1(−) than in combined GSTM1(−)/T1(+) and combined GSTM1(+)/T1(+) (*p* = 0.2 and 0.03, respectively), and that of AST was significantly higher in combination of GSTM1(−)/T1(−) and in combination of GSTM1(+)/T1(−) than in combination of GSTM1(+)/T1(+) (*p* = 10^−3^ and 10^−3^, respectively).

**Conclusions:**

Our findings suggest that the GSTM1 (−) allele may be considered as a key factor for the development of carbamazepine-induced hepatotoxicity. Results related to GSTT (−) allele and elevation in AST levels should be considered with caution as AST may be elevated in other pathophysiological conditions.

## Background

Carbamazepine (CBZ) is an effective drug used for epilepsy, trigeminal neuralgia and many psychiatric disorders [1]. Although considered as a well tolerated anti-epileptic drug, several adverse drug reactions to CBZ may occur including hypersensitivity syndrome, hematotoxicity and hepatotoxicity [2, 3]. Severe CBZ-induced hepatotoxicity is exceptional. Elevation in hepatic enzymes is reported in almost 22% of CBZ-treated patients [4]. Adverse effects to CBZ have been attributed to reactive metabolites of the drug [3, 5]. In fact, in the liver, CBZ is oxidized to its major metabolite, the 10,11-epoxy-carbamazepine (CBZ-E) [6, 7] and to additional metabolites CBZ-arene oxide and CBZ iminoquinone by the cytochrome P450 enzymes [3]. Glutathione S-transferases (GST) are enzymes involved in phase II reactions and playing a key role in detoxifying drugs and xenobiotics. The implication of the metabolites of CBZ has been suggested to induce adverse-drug reactions, cytotoxicity, and also genotoxicity [3, 5].

Both Glutathione S-transferase class mu (GSTM1) and class thêta (GSTT1) are involved in reactions of gluco-conjugation of the main metabolite and also in the detoxification of toxic metabolites of CBZ [3, 7–9]. Null alleles of the GSTM1 and GSTT1 resulting on a deletion of the entire genes, lack any functional enzyme activity and fail to express the protein.. Therefore, functional polymorphisms in the genes of GSTM1 and GSTT1 enzymes may lead to an increased risk for CBZ-induced adverse effects, including hepatotoxicity. Among ethnic groups, GSTM1 and GSTT1 null allele frequencies are varying from 23 to 62% respectively [10–13]. Drug-induced hepatotoxicity is often unpredictable and may result in a transient elevation in transaminase enzymes, but may be fatal leading to acute liver injury. Many reports have investigated the involvment of GSTs null allele in the occurrence of hepatotoxicity with anti-tubercular drugs, in alzheimer’s disease and with troglitazone therapy [14]. In CBZ-induced hepatotoxicity, very few reports have investigated the influence of GSTs alleles in the detoxification of toxic metabolites [14].

To the best of our information, no previous reports in tunisian population nor in subsaharian African population have focused on the effects of GSTM1 and GSTT1 null alleles in elevations of liver enzymes in CBZ-treated patients.

Therefore, in our study, the influence of GSTM1 and GSTT1 null alleles in elevation of transaminase enzyme levels among Tunisian epileptic patients treated with CBZ has been investigated.

## Methods

### Subjects

#### Study design

**Fig. 1:**
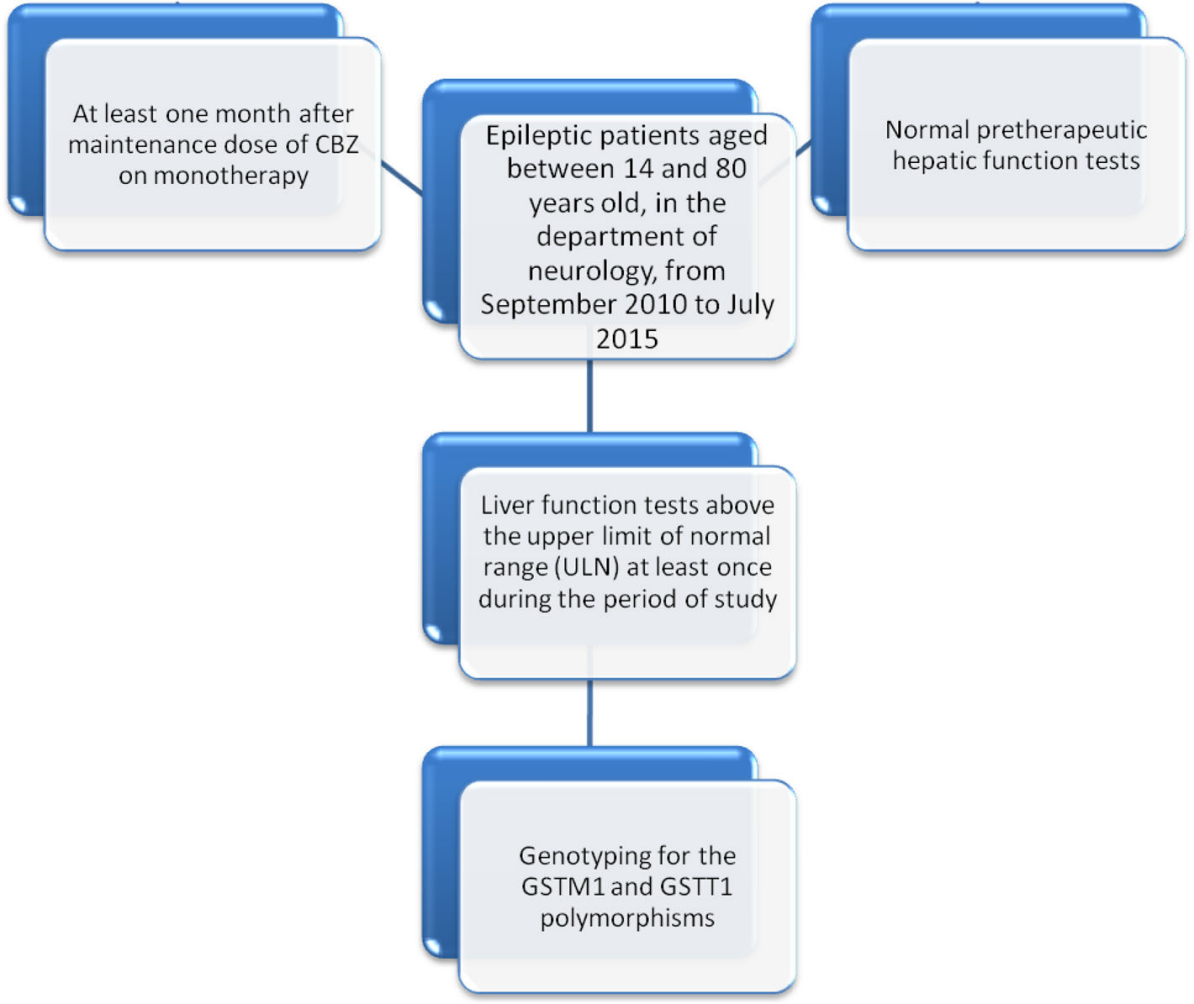
Flow-diagram of the study

A cross-sectional prospective study has been conducted in the Department of Neurology “Sahloul Hospital of Sousse Tunisia”, from September 2010 to July 2015 (Fig. 1).

#### Inclusion criteria

- Epileptic patients aged between 14 and 80 years old on monotherapy by CBZ.
- At least 1 month of obtaining the maintenance dose of CBZ.
- Pretherapeutic liver function tests in normal ranges.
- Hepatotoxicity was defined as raised levels of transaminase above the upper limit of normal range (ULN) at least once during the period of study. Before initiation of CBZ therapy, a complete cell count and hepatic function test were performed. Then, in the first month, two tests were performed once a week and periodically (every 3 months) as recommended [15]. The ULN for aspartate aminotransferase (AST) and alanine aminotransferase (ALT) were 31 UI/L and 34 IU/L respectively.

#### Non-inclusion criteria

- Previous chronic or acute liver disease,
- Presence of serological evidence of infection with hepatitis viruses.
- Chronic renal disease.
- History of chronic alcohol intake and/or herbal consumption.
- Drug consumption interfering with the metabolism of CBZ or with liver function tests.

### Genotyping

Extraction of genomic DNA was performed from blood samples using the Wizard Genomic DNA Purification Kit (cat. *A1120) (Promega, Madison, USA).

The genotyping of GSTM1 and GSTT1 was performed using a previously described Polymerase Chain Reaction (PCR) [16].

In our study, the deleted gene of GSTM1 is termed GSTM1 null allele or GSTM1 (−). Heterozygotes [GSTM1(+/−) and the homozygous wild type, GSTM1(+/+)] are designed GSTM1 (+). As for the GSTT class, deletion of the entire gene is termed GSTT1(−). While heterozygotes [GSTT1(+/−) and the homozygous wild type GSTT1(+/+)] are designed GSTT1 (+).

### Phenotype analysis

An analysis of plasma samples were performed in the Laboratory of Metabolic Biophysics, Professional Toxicology and Applied Environnemental, Department of Biophysics, Medicine Faculty of Sousse, Sousse, Tunisia. Dosage of CBZ and CBZ-E was conducted using previously described high performance liquid chromatography [17].

Serum alanine aminotransferase and aspartate aminotransferase were measured by standard methods adopted by Biochemical Laboratory of University Hospital Sahloul.

### Statistical analysis

The mean values of the ALT and AST in epileptic patients were compared according to the different types of genetic polymorphisms of GST by applying the test U Mann–Whitney. Using the Kolmogorov–Smirnov test to a sample, the variables studied follow a non-normal distribution where recourse to the use of non-parametric test (U-test Mann–Whitney).

The correlation between levels of transaminases and the doses per mg/kg/day or the plasma concentration of carbamazepine were studied by Pearson’s correlation test.

Association between genotype frequencies of GSTM1/T1 allele distribution and carbamazepine-induced mild hepatotoxicity was analyzed by calculating odds ratios (OR) and 95% confidence intervals (CLs). Statistical analysis was performed using the SPSS software package. A probability of *p* < 0.05 was considered statistically significant.

### Ethical considerations

Ethics committee approval and patient consent was obtained for our study.

The local ethical committee of Sahloul University hospital has approved this study. Informations were made anonymous after the collection of the data and blood samples.

## Results

Our study included 129 epileptic Tunisian patients (55 males and 74 females). Almost 54.68% suffered from focal seizures with or without secondary generalization (71 patients), 37.5% had generalized tonic-clonic seizures (48 patients) (Table 1). Patients were on CBZ therapy for 28.16 months (range: 18–36 months). Monitoring for epilepsy was performed for 3 years on average.

**Table 1:** Clinical detail in epileptic patients

Total number of patients studied	129
Seizure type (partial/generalized)	71/48
Epilepsy type N (%) (Symptomatic/Cryptogenic)	48/81
Sex (M/F)	55/74
Age (years)	38.26 ± 17
Weight (kg)	63.3 ± 7.6

In our study, frequency distribution of the null allele of GSTM1 (GSTM1-) and the null allele of GSTT1 (GSTT-) were 74.4 and 17.8%, respectively.

The ALT and AST were elevated in 35.7% (46 cases: 30 males /16 females) and 25.6% (33cases: 22 males / 11 females). The mean levels of ALT and AST was 49 ± 6.44 and 112 ± 10.74, respectively.

A 1 mg/body weight/day increase of CBZ maintenance dosages (mg/kg/day) conferred OR of 1.004 (95% CI: 0.89.-1.12; *p* = 0.95) and 0.99 (95% CI: 0.88–1.12; *p* = 0.91) on the elevation in ALT and AST levels respectively; while a 1 μg/mL increase of plasma carbamazepine concentration conferred OR of 1.062 (95% CI: 0.92–1.22; *p* = 0.40) and 1.14 (95% CI: 0.97–1.33; *p* = 0.11) on the elevation in ALT and AST levels, respectively (Table 2). Neither the dosage of CBZ per body weight nor the plasma CBZ concentrations were associated with the ALT and AST levels. Levels of ALT and AST seem to not be affected by genders and age.

**Table 2:** Relationship between mean dosage of CBZ per body weight, mean plasma CBZ concentrations and ALT/AST levels

	ALT		*p*-value	OR* (95% CI)	AST		*p*-value	OR* (95% CI)
	Normal (n) 83	Elevated (n) 46			Normal (n) 96	Elevated (n) 33		
Mean dosage (mg/kg/day)	7.92 ± 3.80	7.96 ± 2.06	0.94	1.004 (0.89–1.12)	7.96 ± 3.57	7.88 ± 2.26	0.91	0.99 (0.88–1.12)
Mean plasma carbamazepine concentrations (μg/mL)	6.25 ± 2.70	6.65 ± 2.38	0.40	1.062 (0.92–1.22)	6.18 ± 2.77	7.01 ± 1.88	0.11	1.14 (0.97–1.33)

H0: no difference between mean dosage of CBZ per body weight, mean plasma CBZ concentrations and ALT/AST levels

*p*<0.05 is significant

ALT and AST values were significantly higher in GSTM1 null allele (−) than in GSTM1 (+) (*p* = 10^−3^ and 0.004, respectively) (Table 3). Concerning GSTT1, these values were not influenced by GSTT1(−).The results concerning the effects of the combinations of GST genotypes on the corresponding values of transaminases was significantly different (Table 2). The level of ALT was significantly elevated in GSTM1(−)/T1(−) and GSTM1(−)/T1(+) than in GSTM1(+)/T1(+) (*p* = 0.03 and 0.2, respectively). The level of AST was significantly higher in GSTM1 (−)/T1(−) and GSTM1(+)/T1(−) than in GSTM1(−)/T1(+) (*p* = 10^−3^ and 10^−3^, respectively).

**Table 3:** Mean values of ALT and AST in each genotype

	ALT (IU/l)	*p*-value^*^	AST (IU/l)	*p*-value^*^
GSTM1 genotype (n)				
Null (96)	37.5 ± 5,716	**10** ^**−3**^	35.03 ± 11,8	**0.004**
Present (33)	30.93 ± 6,025		28.81 ± 4,35	
GSTT1 genotype (n)				
Null (23)	34.47 ± 8,36	0.27	35.26 ± 8,20	0.37
Present (106)	36.11 ± 5,96		33.05 ± 11,21	
Combination of GSTM1/GSTT1 (n)				
M1+/T1+ (19)	32.74 ± 6,87		28.42 ± 5,57	
M1+/T1- (8)	33.87 ± 10,66		53.62 ± 23,91	**10** ^**-3**a^
M1−/T1+ (66)	35.86 ± 3,88	**0.02** ^a^	29.31 ± 4,57	
M1−/T1- (35)	37.88 ± 8,28	**0.03** ^a^	39.71 ± 8,47	**10** ^**-3**a^

Null hypothese H0: no differences between the mean values of ALT and AST in each GSTM1(−) and GSTT1(−), combined GSTM1(−) and GSTT(−) and mean values of ALT and AST

*p*-value^*^ by Mann-Whitney U test

^a^Compared with M1+/T1+

Bold entries indicate significance

The distribution of GST alleles and the increase of transaminases are shown in Table 4.

**Table 4:** Distribution of GSTM1 and GSTT1 alleles in patients with elevated or normal levels of ALT and AST

	ALT		*p*-value	OR* (95% CI)	AST		*p*-value	OR* (95% CI)
	Normal	Elevated			Normal	Elevated		
GSTM1 genotype (n)								
Null	54	42	**0.0022**	5.64 (1.70–20.60)	64	32	**0.0013**	11 (1.56–77.37)
Present	29	4			32	1		
GSTT1 genotype (n)								
Null	15	8	0.88	0.95 (0.33–2.68)	15	8	0.39	1.73 (0.59–5.01)
Present	68	38			81	25		
Combination of GSTM1/GSTT1								
M1+/T1+	15	4		1.0	17	2		1.0
M1+/T1-	5	3	0.68	0.44 (0.05–3.74)	0	8	0.99	4(Undefined)
M1−/T1+	48	18	0.80	0.71 (0.17–2.73)	64	2	0.45	3.76 (0.34–41.37)
M1−/T1-	14	21	**0.014**	**0.18** (0.04–0.75)	14	21	**0.0012**	**0.08** (0.01–0.45)

Null hypothese (H0): no differences between GSTM1 and GSTT1 alleles in patients with elevated or normal levels of ALT and AST

*p*<0.05 is significant

Bold entries indicate significance

The GSTM1 null genotype frequency was 65.1 and 91.3% in patients with normal and elevated levels of ALT, respectively (OR: 5.64; 95% CI: 1.70–20.60; *p* = 0.0022), and was 66.7 and 97% in cases with normal and elevated levels of AST, respectively (OR: 11; 95% CI: 1.56–77.37; *p* = 0.0013). On the contrary, no difference between the frequency of the GSTT1 (−) in the cases with normal or elevated ALT and AST, respectively was found (OR: 0.95; 95% CI: 0.33–2.68; *p* = 0.88) and (OR: 1.73; 95% CI: 0.59–5.01; *p* = 0.39). So, levels of transaminases were influenced by the genotype GSTM1 (−) and seem to not be affected by GSTT1 (−).

The frequency of GSTM1 (−)/T1 (−) (16.41%) was significantly higher than that of the GSTM1(+)/T1(+) (3.13%) in cases with elevated ALT (OR: 0.18; 95% CI: 0.04–0.75; *p* = 0.014). The frequency of GSTM1 (−)/T1 (−) (16.41%) was also significantly higher than that of the GSTM1(+)/T1(+) (1.56%) in cases with elevated AST (OR: 0.08; 95% CI: 0.01–0.45; *p* = 0.0012).

## Discussion

We performed a study of association between null alleles of GSTM1 and GSTT1 and risk of mild elevation of liver enzymes in Tunisian epileptic patients treated with CBZ on monotherapy. The GSTM1 and GSTT1 null alleles (GSTM1(−) and GSTT1(−)) consisting on a deletion of the complete genes and resulting in a lack of active enzymes are the most common polymorphisms in these genes. The frequencies of GSTM1 and GSTT1 deficiency vary from 23 to 62% respectively [10–12]. In Tunisian population, GSTM1 null allele frequency was of 34.6% and GSTT1 null allele frequency was of 16.6%. The frequency of double deletion of both GSTM1 and GSTT1 was of 4.82% [18].

In another genetic study of the association of GSTM1 and GSTT1 polymorphisms reported in three Arab populations (Bahraini, Lebanese and Tunisian), including 186 Tunisian healthy subjects, the prevalence of GSTT1 null allele was of 37.1%, GSTM1 null allele was of 63.4% and deleted allele of both genes was found in 21.0% of Tunisians [19].

In our population study, the frequency of patients carrying null alleles of GSTM1 and GSTT1 were consistent with these results.

The GSTM1 (−)allele was associated with elevated levels of both ALT and AST. It seems to affect the levels of transaminases in our population study and to be a risk factor for mild hepatotoxicity induced by CBZ. As for the GSTT1 (−) allele, it seems not to influence the ALT and AST levels. Whereas the combination of GSTM1 (+)/GSTT1 (−) alleles seems to affect the levels of AST. In fact, the GSTM1 (−) allele is rather significantly associated with an increase in ALT levels and in AST levels respectively than the GSTT1 (−) allele. Our results are consistent with those of Ueda et al. [20] who reported that GSTM1 (−) allele was related with the risk of mild hepatotoxicity induced by CBZ in Japanese population. As far as we know, this is the first study reporting an association between the GSTM1 (−) allele and the increasing values of transaminases in Tunisian and subsaharian population.

Many reports have associated the GST null alleles, leading to a lack of functional protein, with an increase susceptibility to several diseases, such as cancer, cardiovascular, epilepsy, schizophrenia and hepatotoxicity [16, 21–24]. The reactive metabolites of drugs causing drug toxicity are detoxified by GST enzymes. So, an inherited weakness of drug eliminating capacity related to a null GST alleles may enhance toxic drug-induced adverse events [3, 5, 7, 10, 25]. A 2-fold ULN increased risk of elevation levels of ALT has been reported in individuals exhibiting the GSTM1 (−) allele among patients treated with isoniazid, rifampicin and pyrazinamide for pulmonary tuberculosis [26, 27]. Simon and colleagues [28] showed that 72% of patients carriers of GSTM1(−)/T1(−) alleles have at least three times elevated ALT levels among Alzheimer’s disease. Watanabe and colleagues [27] found higher risk of hepatotoxicity-induced by troglitazone in patients with GSTM1 (−)/T1(−) alleles.

Our study found that the GSTM1 (−) allele, especially when combined with GSTT1 (−) allele, may be a genetic factor associated with the increased risk of hepatotoxicity. In many reports, toxic effects of drugs have been attributed to the formation of chemically reactive metabolites [3, 5]. The metabolites of CBZ, such as 10,11- epoxy-carbamazepine (CBZ-E), arene oxide (s) and iminoquinone (s), may be associated with drug hypersensitivity reactions, cytotoxicity and genotoxicity [3, 5, 7, 25]. The CBZ-E is a chemically reactive metabolite and may be converted into electrophilic reactive intermediate with glutathione (GSH) [26]. So, GST may play a role in the overall covalent binding potential of CBZ-E with liver microsomes and polymorphisms may be responsible for liver-toxicities [29].

In our study, neither the dosage of CBZ per body weight nor the plasma CBZ concentrations were associated with transaminase levels. It is evidence that CBZ-induced hepatotoxicity is dose-independent and carbamazepine toxicity seems to not be correlated with CBZ plasma concentration. It may be rather induced by allergic reactions [3].

GSTM1 seem to affect ALT levels whereas GSTT1 seem to be related to AST levels. These results can be explained, almost partly, by the distribution of transaminases and the GST enzymes. While ALT is mainly distributed in the liver, AST is present in the liver, red blood cells and muscle. Consequently, ALT is therefore considered to be a more accurate sign of liver damage than AST. In addition, GSTM1 is widely expressed in the liver, and GSTT1 is principally expressed in erythrocytes [10, 13]. Consequently, the ALT levels may be more affected by GSTM1 (−) allele while AST may be more susceptible to GSTT1 (−) allele. Thus, our findings suggest that the GSTM1 (−) allele may be considered as a key factor for the development of carbamazepine-induced hepatotoxicity.

However, our findings should be considered with caution. Many limits of our study should be mentioned. First, our population study number is small. Although we tried to be exhaustive and to include all the patients responding to our inclusion criteria, our small sample size just suggests a trend to possible correlation between GSTM1 (−) allele and elevation in ALT and AST levels, additional reports would be indispensable for proving our results in a larger scale.

Second, investigations in patients with elevated AST levels were not performed to rule out other susceptible causes. Moreover, Single laboratory liver test may be of little value.

## Conclusions

An association between GST genotyping and a possible effect in transaminases in an epileptic population study on CBZ in monotherapy was attempted. Our results showed a possible correlation between GSTM1 (−) genotype and elevation in ALT and AST levels. GST null allele may be a risk factor for mild hepatotoxicity in Tunisian epileptic patients. Further studies including a larger sample sizes would be necessary for proving our results.

## Abbreviations

ALT: Alanine aminotransferase
AST: Aspartate aminotransferase
CBZ: Carbamazepine
CBZ-E: 10,11-epoxy-carbamazepine
CLs: Confidence intervals
GSH: Glutathione
GSTM1 (−): Null allele of the GSTM1
GSTM1: Glutathione S-transferase M1
GSTT1 (−): Null allele of the GSTT1
GSTT1: Glutathione S-transferase T1
HPLC: High-performance liquid chromatography
ORs: Odds ratios
PCR: Polymerase Chain Reaction
SD: Standard deviation
ULN: Upper limit of normal range
